# Biothiols and oxidative stress markers and polymorphisms of *TOMM40* and *APOC1* genes in Alzheimer’s disease patients

**DOI:** 10.18632/oncotarget.26184

**Published:** 2018-10-16

**Authors:** Michal Prendecki, Jolanta Florczak-Wyspianska, Marta Kowalska, Jan Ilkowski, Teresa Grzelak, Katarzyna Bialas, Malgorzata Wiszniewska, Wojciech Kozubski, Jolanta Dorszewska

**Affiliations:** ^1^ Laboratory of Neurobiology, Department of Neurology, Poznan University of Medical Sciences, Poznan, Poland; ^2^ Chair and Department of Neurology, Poznan University of Medical Sciences, Poznan, Poland; ^3^ Department of Emergency Medicine, Poznan University of Medical Sciences, Poznan, Poland; ^4^ Division of Biology of Civilization-Linked Diseases, Department of Chemistry and Clinical Biochemistry, Poznan University of Medical Sciences, Poznan, Poland; ^5^ Faculty of Health Care, Stanislaw Staszic University of Applied Sciences in Pila, Pila, Poland; ^6^ Department of Neurology, Specialistic Hospital in Pila, Pila, Poland

**Keywords:** biothiols, oxidative stress, gene polymorphism, Alzheimer’s disease

## Abstract

Alzheimer’s disease (AD) is a progressive disease, with frequently observed improper biothiols turnover, homocysteine (Hcy) and glutathione (GSH). GSH protects cells from oxidative stress and may be determined by 8-oxo-2’-deoxyguanosine (8-oxo2dG) level and its repair enzyme 8-oxoguanine DNA glycosylase (OGG1). The presence of unfavorable alleles, *e.g.,* in *APOE* cluster, *TOMM40* or *APOC1* is known to facilitate the dementia onset under oxidative stress.

The aim of the study was to analyze rs1052452, rs2075650 *TOMM40* polymorphisms, rs4420638 *APOC1*, and their correlation with Hcy, GSH, 8-oxo2dG, OGG1 levels in plasma of AD patients and controls.

We recruited 230 individuals: 88 AD, 80 controls without (UC), 62 controls with (RC) positive family history of AD. The *TOMM40* genotype was determined by HRM and capillary electrophoresis, while *APOC1* by HRM. The concentrations of OGG1, 8-oxo2dG were determined by ELISA, whereas Hcy, GSH by HPLC/EC.

We showed that over 60% of AD patients had increased Hcy levels (p<0.01 *vs.* UC, p<0.001 *vs.* RC), while GSH (p<0.01 *vs.* UC), 8-oxo2dG (p<0.01 *vs.* UC, p<0.001 *vs.* RC) were reduced. Minor variants: rs10524523-L, rs4420638-G, rs2075650-G were significantly overrepresented in AD. For rs4420638-G, rs2075650-G variants, the association remained significant in *APOE* E4 non-carriers. The misbalance of analyzed biothiols, and 8-oxo2dG, OGG1 were more pronounced in carriers of major variants: rs10524523-S/VL, rs4420638-A, rs2075650-A.

We showed, for the first time, that *APOC1* and *TOMM40* rs2075650 polymorphisms may be independent risk factors of developing AD, whose major variants are accompanied by disruption of biothiols metabolism and inefficient removal of DNA oxidation.

## INTRODUCTION

Alzheimer’s disease (AD) is a progressive neurodegenerative disease and a major cause of dementia in adults over 60 years of age, affecting cognitive functions of nearly 24 million patients worldwide [1].

The AD occurs in two forms: familial and sporadic AD [2]. The latter is typical for aging population, and is caused by both, genetic (70%) [3] and environmental (30%) [4] factors. Currently, the pathomechanism of AD is explained in multiple pathways, including dysregulation of the interaction network between the production, clearance, and aggregation of amyloid β (Aβ), a central player in an Aβ cascade hypothesis. This phenomenon may occur more often in individuals with rare variants in genes associated with metabolism of Aβ and cholesterol turnover, located on chromosome 19, in *APOE* gene cluster. The *APOE* gene polymorphisms have been extensively studied, and the emerging research focuses on other genes in the immediate vicinity of the *APOE* gene, *i.e., TOMM40* and *APOC1, APOC2, APOC4* [5–7].

The *APOC2* and *APOC4* are understudied with the last publication for *APOC2* from 2011 and with only a single work for *APOC4* by Cervantes *et al.* [7], who have obtained insignificant results after statistics adjustment. Above genes represent rather modest odds ratios (OR) values for AD [0.82-1.20]. In turn, the *APOC1* gene locus has been reported to house multiple changes deciphered using the long-range sequencing in Caucasians: rs445925, rs59325138, rs390082, rs4420638, rs4803770. These variants exhibit moderate OR values, except rs4420638 (OR=3.46; hereafter *APOC1*’638), which is located 14 kb adjacent to *APOC1* gene [7–9]. The *APOC1* gene encodes apolipoprotein C1, a key regulator of the metabolism of high-density and very-low density lipoproteins [10], and may be associated with cognitive impairment in Chinese [11]. The frequency of ‘638 single nucleotide polymorphism (SNP) in Polish AD patients has not been studied yet.

From the 5’end, the *APOE* gene neighbors with *TOMM40* gene, containing multiple genetic variants described by using the long-range sequencing: N1, rs11556510, rs184017, rs157580, rs2075650, rs157581, rs34095326, rs11556505, rs157582, rs59007384, rs77301115, rs8106922, N5, rs73052321, rs741780, rs405697, rs10119 [7]. Most of these variants are located deep in intronic sequences and should have little effect on splicing machinery, except rs2075650 (hereafter *TOMM40*’650), which sits just 31bp from exon junction [12]. The reported OR for this SNP is 2.94 [CI 2.31-3.74], and minor allele frequency is 0.214 [7]. The association of *TOMM40*‘650 with AD has been confirmed by two independent mataanalyses [13, 14]. Moreover, aside AD, the ‘650 SNP was shown to be associated with a decreased delayed recall score in persons above 60 years of age [15]. For Polish population, no data has been reported to date.

Another frequently studied variant in *TOMM40* is a highly polymorphic poly-T fragment in the intron 6, rs10524523 (hereafter ‘523), with differing number of deoxythymidine repeats. Three “alleles” of ‘523 polymorphism have been described: Short (S, ≤19 T), Long (L, 20-29 T) and Very Long (VL, ≥30 T) with ability to impact the age of onset (AOO) of AD, as well as the brain structure and cognitive functions [16–26]. The effect of ‘523 polymorphism on AOO of Polish AD patients has been analyzed only in one study [24], which did not correlate this SNP with other variants in *APOE* cluster beside E2/E3/E4. The *TOMM40* gene encodes translocase of the outer mitochondrial membrane homolog 40 (TOM40). In AD, TOM40 channels may be blocked by Aβ, and this enables Aβ to enter the mitochondria, and the accumulation of Aβ in mitochondria leads to the overproduction of reactive oxygen species (ROS) [27, 28]. The accumulation of ROS might be regulated by natural antioxidants, including glutathione (GSH).

The GSH in mitochondria (mtGSH) inhibits intramitochondrial proteins form entering the cytoplasm and inducing the cell death machinery. The mtGSH forms an alternative pool to cytoplasmic GSH and requires specific carriers to enter the mitochondria, such as TOM40 [29]. The blockade and underexpression of TOM40 lead to an impaired penetration of GSH into the mitochondria, which in turn causes the excessive ROS production, mitochondrial failure and releasing of caspases – the apoptosis inducers, contributing to progressive neuronal loss and the onset of dementia [30]. Moreover, in the course of the several neurodegenerative diseases, including AD, the GSH concentration may be decreased. Hence the GSH has been proposed as a biomarker for diagnosis of neurodegenerative diseases, and promising target of future therapies [31]. Especially helpful would seem the increasing of the mtGSH pool, due to its protective role to the mitochondria [32].

The regulation of mtGSH may also be misbalanced by the accumulation of homocysteine (Hcy), a metabolite of methionine (Met) [33]. Furthermore, the high concentration of Hcy would impede production of GSH, since 50% is metabolized back to Met, and the rest to cysteine (Cys), subsequently used for GSH synthesis [34]. Hcy may directly inhibit the mtGSH pool renewal, exhibiting pronounced toxicity to isolated rodent hippocampal mitochondria. Moreover, numerous studies have confirmed the increased plasma concentration of Hcy as a risk factor of developing dementia and vascular diseases [35].

The high levels of Hcy (hyperhomocysteinemia, HHcy) may result in increased production of ROS in mitochondria, followed by formation of 8-oxo-2’-deoxyguanosine (8-oxo2dG). The accumulation of 8-oxo2dG in the DNA arises early the course of AD, and may advance with disease progression [36]. The concentration of 8-oxo2dG is elevated in the brain cells as well as in the DNA of peripheral lymphocytes or serum of AD patients [37]. The presence of 8-oxo2dG in DNA during replication may lead to inordinate base pairing, causing somatic mutations and loose of genes functions in up to 14% of events [38]. In order to prevent mispairing, the 8-oxo2dG is removed from DNA by a repair enzyme: 8-oxoguanine DNA glycosylase (OGG1), a protein associated with base excision repair (BER) system, depending on removal of modified nucleotides from DNA before replication and fixation of the mutation [39]. The concentration of OGG1 in serum and lymphocytes isolated from AD patients may be decreased in the course of the disease [37, 40], and the mRNA expression of certain isoforms of *OGG1* are significantly dysregulated in AD, and therefore may impede the OGG1 function and serve as AD biomarker [41].

Importantly, the OGG1 enzyme similarly to GSH requires TOM40 machinery to enter the mitochondria. According to the literature, the *TOMM40*’523 poly-T variants may influence the amount of TOM40 and hence affect the regulation of oxidative damage scavengers and repair mechanisms [42–45]. The physiological level of OGG1 and GSH may protect the cell against oxidative damage, and affect the onset and progression of AD. It seems that the oxidative stress in AD may be associated with genes in *APOE* cluster, which may undergo expression regulation by PPARγ [46], a transcription factor associated with a reduction of oxidative stress in mitochondria [47].

In turn, the involvement of *APOC1*’638 and *TOMM40*’650 SNPs in the generation of oxidative stress in AD remains unknown. There is also no information on how each of these variants influences the level of biochemical factors associated with oxidative stress in AD.

The aim of the study was to analyze the plasma concentration of biothiols: Hcy, GSH as well as the concentration of a product of oxidative DNA damage: 8-oxo2dG, and the concentration of the OGG1, a repair enzyme that is able to remove oxidized guanosine from the DNA. Furthermore, we analyzed the correlation between polymorphisms of three loci within *APOE* gene cluster, *i.e.* two located in proximity of *TOMM40*: a polyT length polymorphism in intron 6 (‘523) and SNP in intron 2 (‘650), as well as SNP adjacent to *APOC1* gene: rs4420638 (‘638), and concentration of measured biochemical parameters in AD patients, related and unrelated controls in Polish population.

## RESULTS

### Biochemical parameters

Our study has shown a significant increase in Hcy concentration in AD patients as compared to both, UC and RC (p<0.01 and p<0.001, respectively), as shown in Table 1. The elevated plasma Hcy (hyperhomocysteinemia, Hcy level above 15 μmol/L) we observed in 60.9% AD patients as compared to 34.2% of UC and 32.3% of RC (OR=3.00, 95% CI: 1.53-5.86, p<0.01 and OR=3.27, 95% CI: 1.59-6.71, p<0.01, Fisher exact test, respectively). The highest median Hcy concentration was observed in persons with possible preclinical AD (mild cognitive impairment, MCI; 24-27 points in MMSE scale; Hcy 20.3 μmol/L) and in AD patients before the pharmacotherapy with common AD drugs (Hcy 22.9 μmol/L, p<0.01 *vs.* UC and p<0.001 *vs.* RC). Subsequently, the lowest median concentration of Hcy was found in AD patients with moderate dementia (11-18 MMSE; Hcy 16.0 μmol/L) or treated with AchEI (donepezil, rivastigmine; Hcy 14.9 μmol/L), the detailed data is shown in Supplementary Tables 1 and 2.

**Table 1:** The concentration of homocysteine (Hcy), glutathione (GSH), 8-oxo-2’-deoxyguanosine (8-oxo2dG) and 8-oxoguanine DNA glycosylase (OGG1) in plasma of Alzheimer’s disease (AD) patients and related controls (RC) and unrelated controls (UC)

Parameters	Unrelated controls (UC)	Related controls (RC)	Alzheimer’s disease (AD)	p			
				AD *vs.* UC *vs.* RC	AD *vs.* UC	AD *vs.* RC	UC *vs.* RC
**Hcy [μmol/L]**	13.1 [10.6-17.5]	13.2 [10.8-16.5]	18.1^**(***)^ [12.1-21.2]	**p=0.0007**^#^	**p<0.01**^$^ **p=0.0012**^@^	**p<0.001**^$^ **p=0.0008**^@^	p>0.05^$^ p=0.8697^@^
**GSH [μmol/L]**	910.0 [782.5-1116]	887.1 [764.6-1055]	790.0^**^ [691.1-1039]	**p=0.0459**^#^	**p<0.01**^$^ **p=0.0084**^@^	p>0.05^$^ p=0.0511^@^	p>0.05^$^ p=0.2090^@^
**GSH/Hcy**	73.0 [52.2-88.6]	68.5 [53.4-82.7]	49.0^***(***)^ [38.0-67.8]	**p<0.0001**^#^	**p<0.001**^$^ **p<0.0001**^@^	**p<0.001**^$^ **p=0.0004**^@^	p>0.05^$^ p=0.7463^@^
**8-oxo2dG [ng/mL]**	5.016 [1.576-7.081]	6.284^***^ [4.956-8.692]	1.919^**(***)^ [0.9960-4.366]	**p<0.0001**^#^	**p<0.01**^$^ **p=0.0052**^@^	**p<0.001**^$^ **p=0.0001**^@^	**p<0.01**^$^ **p=0.0030**^@^
**OGG1 [ng/mL]**	1.211 [0.5765-2.101]	1.706^**^ [1.002-2.503]	1.400 [0.7390-2.194]	**p=0.0293**^#^	p>0.05^$^ p=0.2206^@^	p>0.05^$^ p=0.1142^@^	**p<0.01**^$^ **p=0.0099**^@^
**8-oxo2dG/OGG1**	3.257 [1.635-6.204]	3.298 [1.868-6.170]	1.586^**(***)^ [0.6464-3.298]	**p=0.0006**^#^	**p<0.01**^$^ **p=0.0013**^@^	**p<0.001**^$^ **p=0.0006**^@^	p>0.05^$^ p=0.7423^@^

Median [1^st^ - 3^rd^ quartile]; ^#^-Kruskal-Wallis test, ^$^-followed by Dunn’s Multiple Comparisons test ^@^-Mann-Whitney test; ^**^p<0.01, ^***^p<0.001, as compared to unrelated controls, (^***^) p<0.001 as compared to related controls.

For the biochemical studies, the minimal number of cases to achieve desired sample power (0.800) was estimated for 42 cases by *T* test. Therefore, the number of patients included to the present study provided sufficient sample power (0.9560). Due to the significant differences in Hcy levels in studied groups, affecting other parameters, sample power was not calculated for remaining parameters.

Subsequently, we found that in the plasma of AD patients, the concentration of GSH was significantly reduced as compared with the UC (p<0.01) and the decrease was close to significant as compared to RC (p=0.0511), as shown in Table 1. Moreover, the level of GSH changed with the progress of the disease (Supplementary Table 1). We observed the lowest median level of GSH in MCI (GSH 691.0 μmol/L) and in severe AD (0-10 MMSE; GSH 772.0 μmol/L, p<0.05 *vs.* UC), accompanied by elevated Hcy levels, while the highest GSH was measured in mild and moderate AD (GSH 832.5 μmol/L, p<0.05 *vs.* UC and GSH 822.5 μmol/L, respectively), where Hcy concentrations were less heightened. The change of GSH concentration was significant in the mild and severe AD (p<0.05 *vs.* UC). Subsequently, the AD patients treated with AchEI or AchEI and memantine had lowered median GSH (GSH 772.0 μmol/L, p<0.01 *vs.* UC, p<0.05 *vs.* RC and 755.5 μmol/L), while treatment with memantine alone elevated plasma GSH (GSH 1091.5 μmol/L, *vs.* RC p<0.05; Supplementary Table 2). Additionally, we observed that GSH level was decreased with the duration of AD both lower and over five years (GSH 828.5 and 778.0 μmol/L, p<0.05 *vs.* UC), however with similarly elevated Hcy concentration (p<0.05 *vs.* UC and RC; Supplementary Table 3).

We also assessed the plasma concentration of oxygenated guanosine excised from DNA (8-oxo2dG), and the excising enzyme (OGG1). Our studies indicated a significant reduction of median 8-oxo2dG concentration in AD patients, as compared to UC and RC (p<0.01 and p<0.001, respectively, see Table 1). The decrease was most visible in mild dementia (1.282 ng/mL; p<0.01 *vs.* UC and p<0.001 *vs.* RC; Supplementary Table 1), and was accompanied by the relatively high level of plasma GSH (as described above). A reverse trend was observed in RC whose decreased GSH was accompanied by increased 8-oxo2dG (p<0.01 *vs.* UC), as well as in MCI, severe dementia and AD lasting over five years.

The concentration of OGG1 was significantly increased in RC (p<0.01) and insignificantly in AD (p=0.2206) as compared with UC. The OGG1 increase in RC was accompanied with respective 8-oxo2dG rise as compared to UC, what explains similar median 8-oxo2dG/OGG1 ratios (3.257 *vs.* 3.298). Moreover, significantly worse efficiency of OGG1 dependent BER mechanism (lowest ratio of 8-oxo2dG/OGG1) was observed in AD patients before the treatment (0.8072, p<0.05 *vs.* UC and RC), in the first five years of AD (1.379, p<0.01 *vs.* UC and p<0.001 *vs.* RC), in patients with mild and moderate dementia (0.7233, p<0.001 *vs.* UC and RC, and 1.846, p<0.05 *vs.* RC; Supplementary Tables 1-3).

### Genetic analysis

The frequency of all genotypes is shown in Table 2. The minor alleles: *TOMM40*’523-L,’650-G and *APOC1*’638-G were shown to be significantly overrepresented in AD group as compared to controls (‘523 L *vs.* S+VL: OR=5.61, 95% CI: 2.87-10.9, p<0.0001; ‘650 G *vs.* A: OR=6.12, 95% CI: 2.98-12.6, p<0.0001; and ‘638 G *vs.* A: OR=5.66, 95% CI: 3.07-10.4, p<0.0001), and were more specific than *APOE* E4 (OR=5.14, 95% CI: 2.68-9.85, p<0.0001). The significant differences between AD and RC groups were observed only for *TOMM40*’650 and *APOC1*’638 SNPs (‘650 G *vs.* A: OR=2.12, 95% CI: 1.19-3.78, p<0.05; and ‘638 G *vs.* A: OR=1.76, 95% CI: 1.06-2.92, p<0.05; Table 2). Subsequently, 78.4% of AD patients were carriers of at least one of studied alleles, as compared to 30.0% of UC and 56.5% of RC (OR=8.47, 95% CI: 4.21-17.0, p<0.0001 and OR=2.80, 95% CI: 1.37-5.72, p<0.01; p values were calculated with Fisher exact test).

**Table 2:** Alzheimer’s disease and control’s *APOE*, *TOMM40* and *APOC1* minor allele frequencies (MAF) and genotypes

Minor alleles	Unrelated controls (UC)	Related controls (RC)	Alzheimer’s disease (AD)		AD *vs.* UC	AD *vs.* RC	UC *vs.* RC
*APOE* E4 [%]	8.10	24.2^***^	31.3^***^	OR	5.14	1.42	3.61
				95% CI	2.68-9.85	0.85-2.40	1.79-7.27
				p	**<0.0001**	0.1953	**0.0002**
*TOMM40*‘523-L [%]	7.50	26.2^***^	31.3^***^	OR	5.61	1.28	4.39
				95% CI	2.87-10.9	0.765-2.14	2.15-8.95
				p	**<0.0001**	0.3672	**<0.0001**
*TOMM40*‘650-G [%]	6.20	16.1^*^	29.0^***(*)^	OR	6.12	2.12	2.88
				95% CI	2.98-12.6	1.19-3.78	1.30-6.42
				p	**<0.0001**	**0.0127**	**0.0105**
*TOMM40*‘650-G [%] (in *APOE* E4 noncarriers)	5.20	7.40	23.8^***(**)^	OR	5.65	3.92	1.43
				95% CI	2.25-14.2	1.38-1.42	0.4395
				p	**0.0001**	**0.0074**	4.7177
*APOC1*‘638-G [%]	9.40	25.0^***^	36.9^***(*)^	OR	5.66	1.76	3.22
				95% CI	3.07-10.5	1.06-2.92	1.65-6.29
				p	**<0.0001**	**0.0328**	**0.0006**
*APOC1*‘638-G [%] (in *APOE* E4 noncarriers)	3.70	2.90	13.8^*(*)^	OR	4.11	5.26	0.782
				95% CI	1.37-12.3	1.12-24.6	0.147-4.14
				p	**0.0131**	**0.0221**	1.0000
(‘523-L)+(‘650-G)+(‘638-G) [%]	7.70	22.4^***^	33.6^***(***)^	OR	6.07	1.75	3.46
				95% CI	4.15-8.88	1.30-2.37	2.29-5.24
				p	**<0.0001**	**0.0003**	**<0.0001**

GENOTYPES					
Locus	Variant	UC	RC	AD	p^#^
*TOMM40*‘523 genotypes [%]	S/S	21.3	4.9	8.0	**0.0002**
	S/L	11.3	21.3	23.8	
	S/VL	38.8	29.5	25.0	
	L/L	0.0	6.6	11.4	
	L/VL	3.8	18.0	15.9	
	VL/VL	25.0	19.7	15.9	
*TOMM40* ‘650 genotypes [%]	A/A	87.5	74.2	55.7	**0.0001**
	A/G	12.5	19.4	30.7	
	G/G	0.0	6.4	13.6	
*APOC1*‘638 genotypes [%]	A/A	81.3	53.2	39.8	**<0.0001**
	A/G	18.7	43.6	46.6	
	G/G	0.0	3.2	13.6	

percent values; Fisher exact test, ^*^p<0.05, ^**^p<0.01, ^***^p<0.001 as compared to unrelated controls, (^*^/^**^/^***^) p as compared to related controls, p^#^-Chi squared test.

Moreover, the rare variants were also more frequent in persons with *APOE* E4 genotype. However, the observed linkage was not perfect. The highest coinheritance with *APOE* E4 was shown for the *TOMM40’523* L genotype, where 92.3% of UC, 100% of RC and 92.7% of AD patients were heterozygous for both *APOE* E4 and *TOMM40* L alleles. The similar, but the weaker trend was observed for *APOC1*’638 polymorphism, where 76.9% of UC, 92.4% of RC and 75.6% of AD patients carried one copy of *APOE* E4 and *APOC1*’638 G alleles. On the contrary, the linkage between *APOE* E4 and *TOMM40*’650 was much less pronounced, since only 23.1% of UC, 23.1% of RC and 26.8% of AD cases have inherited the heterozygous *TOMM40*’650 G allele together with *APOE* E4 (as shown in Table 2, 3). We also performed a simple *in silico* analysis of the effect of *TOMM40*’650 minor variant on splicing machinery, and we found a new potential donor splice site: GGGgtggag, exciding the threshold consensus value (65) for the Human Splicing Finder algorithm ver. 3.1 [48].

**Table 3:** The frequency of *TOMM40*‘523, *TOMM40*’650 and *APOC1*’638 polymorphisms in relation to *APOE* E4 alleles

*APOE* groups		Unrelated controls (UC)			Related controls (RC)			Alzheimer’s disease (AD)		
Genotypes of *TOMM40*&*APOC1*		EX/EX	EX/E4	E4/E4	EX/EX	EX/E4	E4/E4	EX/EX	EX/E4	E4/E4
*TOMM40*’523 Genotypes [%]	S/S	23.9	7.7	-	9.1	-	-	17.5	-	-
	S/VL	46.2	-	-	54.5	-	-	47.5	7.3	-
	VL/VL	29.9	-	-	36.4	-	-	35.0	-	-
	S/L	-	69.2	-	-	50.0	-	-	51.2	-
	L/L	-	-	-	-	7.7	100	-	9.8	85.7
	L/VL	-	23.1	-	-	42.3	-	-	31.7	14.3
*TOMM40*’650 Genotypes [%]	A/A	89.6	76.9	-	85.3	65.4	-	57.5	61.0	14.3
	A/G	10.4	23.1	-	14.7	23.1	50.0	37.5	26.8	14.3
	G/G	-	-	-	-	11.5	50.0	5.0	12.2	71.4
*APOC1*’638 Genotypes [%]	A/A	92.5	23.1	-	94.1	3.8	-	75.0	12.2	-
	A/G	7.5	76.9	-	5.9	92.4	50.0	22.5	75.6	14.3
	G/G	-	-	-	-	3.8	50.0	2.5	12.2	85.7

percent values; EX – *APOE* E2 or E3; E4 – *APOE* E4.

We observed a significant AOO effect for *TOMM40*’523 polymorphism. The most visible difference was between S/S and L/L *vs.* VL/VL genotypes, where S/S carriers developed disease 7.9±7.5 years earlier than patients with VL/VL genotype (p<0.05, unpaired *T* test), whereas L/L carriers 7.8±5.4 years earlier (p<0.01, unpaired *T* test). The *TOMM40*’650 G/G genotype carriers had significantly earlier AOO than A/G carriers (4.6±4.5 years, p<0.05, unpaired *T* test with Welch correction), while the difference between A/A and G/G was not significant. Similarly, the *APOC1*’638 caused earlier AOO only in G/G carriers (5.1±4.1 years, p<0.05 *vs.* A/A and 5.1±3.3 years, p<0.01 *vs.* A/G, unpaired *T* test with Welch correction).

In turn, the AD patients with aggregation of minor alleles: *TOMM40*’523 L/L, ‘650 G/G, and *APOC1*’638 G/G exhibited 13.7±6.9 years earlier AOO than those with major variants: *TOMM40* VL/VL, A/A and *APOC1* A/A (p<0.01, unpaired *T* test). The cumulative effect of three polymorphisms was much stronger than the effect of *APOE* E4 allele alone since the E4/E4 carriers showed just 7.2±6.9 years earlier onset than E4 non-carriers (p<0.05, unpaired *T* test), see Supplementary Table 4.

### TOMM40 and APOC1 genotypes and biochemical parameters

The analysis also included the assessment of the concentrations of Hcy, GSH, 8-oxo2dG and OGG1 in relation to genetic variants of *TOMM40*’523, *TOMM40*‘650, and *APOC1*’638 SNPs.

We have found that the AD patients with *TOMM40*‘523 S/S showed a nonsignificant tendency to increased and L/VL significant elevated (p<0.05 *vs.* RC) Hcy levels as compared to controls, accompanied by decreased GSH concentration. The highest GSH in UC was observed in *TOMM40*’523 S/L carriers, while in AD in L/L carriers. In both, UC and RC with VL/VL genotype the GHS level was slightly elevated as compared to median for all genotypes, however, the AD patients with VL/VL genotype showed the lowest GSH level (p<0.01 *vs.* UC and p<0.05 *vs.* RC), accompanied by slightly elevated Hcy level, and decreased GSH/Hcy ratio (p<0.01 *vs.* UC and p<0.05 *vs.* RC). The difference in median OGG1 plasma level was most significant in *TOMM40’523* S/S genotype carriers (p<0.01 *vs.* UC), where the concentration of 8-oxo2dG was almost 3-fold lower as compared to RC (p<0.01) and was accompanied by a significant decrease of the 8-oxo2dG/OGG1 ratio (p<0.05 *vs.* UC). On the contrary, in AD patients with VL/VL genotype, the 8-oxo2dG was significantly reduced and accompanied by a reduction of OGG1 and 8-oxo2dG/OGG1 ratio, as compared to UC (Table 4).

**Table 4:** The concentration of homocysteine (Hcy), glutathione (GSH), 8-oxo-2’-deoxyguanosine (8-oxo2dG) and 8-oxoguanine DNA glycosylase (OGG1) in plasma of Alzheimer’s disease (AD) patients, related (RC) and unrelated controls (UC) stratified according to *TOMM40*’523 genotype

*TOMM40*’523	Unrelated controls (UC)						Related controls (RC)						Alzheimer’s disease (AD)					
Parameters	S/S	S/L	S/VL	L/L	L/VL	VL/VL	S/S	S/L	S/VL	L/L	L/VL	VL/VL	S/S	S/L	S/VL	L/L	L/VL	VL/VL
Hcy [μmol/L]	12.6	15.3	13.6	-	11.6	12.2	11.0	12.7	14.0	11.6	13.6	12.6	20.5	16.9	13.8	16.0	**20.0**^(*)^	18.1
	[9.53-16.1]	[11.0-16.0]	[11.1-18.4]		[10.9-49.1]	[9.87-15.1]	[10.8-21.4]	[10.4-16.1]	[11.3-16.6]	[9.91-20.1]	[10.3-15.9]	[10.7-17.0]	[17.3-25-8]	[11.7-22.4]	[11.4-22.9]	[14.1-19.79]	[16.0-21.0]	[10.9-19.3]
GSH [μmol/L]	968.0	1028	871.2	-	874.2	959.5	776.0	922.0	847.5	872.7	965.0	939.5	780.0	867.0	790.0	1067	799.0	731.0^**(*)^
	[795.0-1134]	[825.0-1168]	[771.2-991.0]		[571.0-913.0]	[807.0-1160]	[632.0-1073]	[864.6-997.0]	[731.0-962.0]	[723.0-1097]	[753.8-1059]	[784.9-1067]	[779.2-861.4]	[742.7-996.5]	[651.0-1023]	[681.1-1166]	[672.0-1098]	[681.1-828.7]
GSH/Hcy	76.79	72.99	66.53	-	75.37	79.65	58.53	76.17	63.04	73.85	58.45	69.96	42.83	58.25	54.42	60.28	**45.95**^(*)^	41.89^**(*)^
	[64.07-104.4]	[62.16-86.01]	[50.27-79.91]		[11.63-83.79]	[61.42-101.6]	[50.13-70.23]	[64.4-83.9]	[52.21-80.85]	[53.68-82.64]	[52.31-109.5]	[56.86-86.80]	[30.25-66.59]	[42.60-70.74]	[37.53-65.00]	[38.82-80.84]	[41.51-57.04]	[37.63-59.23]
8-oxo2dG [ng/mL]	5.482	1.675	5.016	-	1.426	4.750	8.232	5.799	6.306	5.299	6.763	8.036^*^	2.796	1.412	**2.308**^(**)^	1.691	**2.839**^(*)^	1.554
	[3.177-7.965]	[0.7820-9.117]	[1.898-6.845]		[1.223-21.61]	[0.9770-6.528]	[6.974-9.490]	[2.526-8.382]	[5.783-8.259]	[3.376-10.241]	[6.048-8.185]	[4.974-13.90]	[2.246-3.298]	[0.9770-3.525]	[1.059-4.346]	[0.7760-2.705]	[0.9960-5.212]	[1.210-12.21]
OGG1 [ng/mL]	1.186	0.9450	1.292	-	6.826	1.162	1.095	1.857^*^	1.750	2.111	1.705	1.419	2.967^**^	1.318	1.502	1.403	1.399	0.617
	[0.4935-1.789]	[0.5180-1.202]	[0.7430-2.104]		[0.4440-10.50]	[0.6185-2.161]	[0.7130-1.477]	[0.9760-2.531]	[1.022-2.636]	[1.723-10.50]	[1.063-2.193]	[0.5280-2.981]	[2.956-4.158]	[0.7600-1.689]	[1.116-2.019]	[1.018-2.370]	[0.8350-2.057]	[0.4490-1.125]
8-oxo2dG/OGG1	5.340	2.531	3.257	-	2.058	2.858	9.016	3.246	3.469	1.599	3.298	4.881	0.946^*^	1.475	2.047	1.282	2.333	1.203
	[1.928-6.741]	[1.081-9.669]	[1.660-6.107]		[0.2089-2.755]	[1.4268-4.719]	[4.722-13.310]	[2.375-4.962]	[1.873-4.972]	[0.9753-3.075]	[2.632-6.811]	[1.659-41.61]	[0.7932-1.118]	[0.6053-2.675]	[0.6566-2.906]	[0.4203-3.277]	[1.531-3.298]	[0.8073-27.18]

Median [1^st^-3^rd^ quartile]; Mann-Whitney test, ^*^p<0.05, ^**^p<0.01, as compared to unrelated controls; (^*^/^**^) p as compared to related controls.

Subsequently, the analysis of *TOMM40*’650 SNP showed that in AD patients with A/A genotype the level of Hcy was significantly increased (p<0.01 *vs.* UC and RC), while GSH/Hcy ratio (p<0.001 *vs.* UC and p<0.01 *vs.* RC), 8-oxo2dG (p<0.05 *vs*. UC and p<0.001 *vs.* RC) and 8-oxo2dG/OGG1 ratio (p<0.01 *vs.* UC and RC) were significantly reduced, as compared to controls with the same genotype, regardless of family history of AD. Furthermore, we observed a tendency for decreased median GSH level in plasma of AD patients with G/G genotype as compared with RC with the same genotype. In both, RC and in AD, the ratio of GSH/Hcy tended to decrease with the presence of *TOMM40*’650 G allele in dose dependent manner. The reduction was more pronounced in RC. However, the lower values were observed for AD patients, as shown in Table 5. No similar trends were observed for other parameters.

**Table 5:** The concentration of homocysteine (Hcy), glutathione (GSH), 8-oxo-2’-deoxyguanosine (8-oxo2dG) and 8-oxoguanine DNA glycosylase (OGG1) in plasma of Alzheimer’s disease (AD) patients and related (RC) and unrelated controls (UC), stratified according to *TOMM40*’650 genotype

*TOMM40*	Unrelated controls (UC)			Related controls (RC)			Alzheimer’s disease (AD)			p			
Parameter	A/A	A/G	G/G	A/A	A/G	G/G	A/A	A/G	G/G		AD *vs.* UC	AD *vs.* RC	UC *vs.* RC
Hcy [μmol/L]	13.05	15.30	-	13.10	12.95	13.35	18.30^**(**)^	15.95	18.50	A/A A/G G/G	**0.0028** 0.3751 -	**0.0034** 0.1715 0.5035	0.9775 0.6016 -
	[10.60-18.23]	[11.9-16.0]		[10.7-16.8]	[11.08-15.70]	[10.54-20.70]	[12.2-21.9]	[11.2-19.7]	[14.1-25.3]				
GSH [μmol/L]	916.0	894.0	-	901.1	823.0	917.2	805.0	809.0	691.0	A/A A/G G/G	0.6669 0.0727 -	0.9879 0.1715 0.5931	0.5607 0.5671 -
	[784.1-1099]	[782.5-1118]		[770.0-1045]	[745.0-1032]	[695.5-1122]	[705.5-1067]	[655.3-906.4]	[581.0-1140]				
GSH/Hcy	72.62	72.99	-	69.92	66.75	52.12	49.00^***(**)^	45.90	43.38	A/A A/G G/G	**0.0004** 0.0951 -	**0.0022** 0.0531 0.4140	0.6234 0.9170 -
	[52.21-94.52]	[60.76-77.69]		[52.31-83.92]	[59.74-76.57]	[44.14-97.29]	[40.68-66.59]	[33.76-73.89]	[36.82-69.28]				
8-oxo2dG [ng/mL]	5.220	3.023	-	6.396^*^	5.709^**^	6.631	2.111^*(***)^	**1.617**^(*)^	2.125	A/A A/G G/G	**0.0115** 0.6436 -	**0.0002** **0.0125** 0.0503	**0.0192** **0.0036** -
	[1.620-7.404]	[1.446-4.124]		[4.974-9.490]	[4.937-8.311]	[4.754-7.657]	[0.9960-5.212]	[0.9410-4.325]	[1.072-4.166]				
OGG1 [ng/mL]	1.273	0.590	-	1.640	2.002*	2.330	1.397	1.436	1.328	A/A A/G G/G	0.4204 0.1961 -	0.4078 0.1248 0.2091	0.1059 **0.0409** -
	[0.6185-2.101]	[0.4945-1.869]		[0.9240-2.464]	[1.015-2.636]	[2.111-3.442]	[0.7600-2.120]	[0.7020-2.194]	[0.6850-2.257]				
8-oxo2dG/OGG1	3.257	4.022	-	3.102	3.075	2.378	1.705^**(**)^	1.118	3.102	A/A A/G G/G	**0.0078** 0.1961 -	**0.0050** 0.0682 0.7273	0.4778 0.9678 -
	[1.635-6.050]	[1.332-6.486]		[1.659-6.777]	[1.873-4.789]	[1.599-2.632]	[0.6464-3.298]	[0.6294-3.496]	[0.8072-3.277]				

Median [1^st^-3^rd^ quartile]; Mann-Whitney test; ^*^p<0.05, ^**^p<0.01, ^***^p<0.001 as compared to unrelated controls, (^*^/^**^/^***^) p as compared to related controls.

Our analysis on *APOC1*’638 SNP showed that in AD patients with A/A genotype, the GSH content (p<0.01 *vs.* UC), GSH/Hcy ratio (p<0.01 *vs*. UC and RC), 8-oxo2dG level (p<0.05 *vs*. UC and p<0.001 RC) and 8-oxo2dG/OGG1 ratio (p<0.01 *vs.* UC and RC) were significantly reduced, as compared to controls with or without family history of AD. Subsequently, for A/G genotype carriers, we observed significant increase of median Hcy level (p<0.01 *vs.* UC and p<0.001 *vs.* RC), accompanied by reduction of GSH/Hcy ratio (p<0.01 *vs.* UC and RC) and decreased of 8-oxo2dG level (p<0.01) and increased OGG1 content (p<0.01), followed by reduced 8-oxo2dG/OGG1 ratio (p<0.05), as compared to RC. Additionally, in controls with the G allele, we observed a tendency for decrease of GSH concentration. In turn, the reverse trend was found in RC. In both, UC and RC carriers of *APOC1*’638 G allele, we observed a tendency for dose dependent decrease of 8-oxo2dG/OGG1 ratio (Table 6).

**Table 6:** The concentration of homocysteine (Hcy), glutathione (GSH), 8-oxo-2’-deoxyguanosine (8-oxo2dG) and 8-oxoguanine DNA glycosylase (OGG1) in plasma of Alzheimer’s disease (AD) patients and related (RC) and unrelated controls (UC), stratified according to *APOC1*’638 genotype

*APOC1*	Unrelated controls (UC)			Related controls (RC)			Alzheimer’s disease (AD)			p			
Parameters	A/A	A/G	G/G	A/A	A/G	G/G	A/A	A/G	G/G		AD *vs.* UC	AD *vs.* RC	UC *vs.* RC
Hcy [μmol/L]	13.15	11.60	-	13.20	12.70	19.45	17.25	19.05^**(***)^	14.40	A/A A/G G/G	0.1680 **0.0019** -	0.1688 **0.0006** 0.9091	0.8790 0.8234 -
	[10.87-18.40]	[10.40-15.30]		[11.07-16.55]	[10.29-15.90]	[10.90-28.00]	[10.90-22.90]	[14.70-21.20]	[12.30-19.79]				
GSH [μmol/L]	914.5	874.2	-	878.0	922.0	947.0	779.5^**^	881.0	828.0	A/A A/G G/G	**0.0042** 0.7452 -	0.0613 0.6203 0.5818	0.3896 0.9791 -
	[794.4-1088]	[732.6-1167]		[764.6-1068]	[775.0-1020]	[709.0-1185]	[681.1-920.3]	[722.9-1054]	[681.1-1140]				
GSH/Hcy	70.61	75.37	-	69.61	68.59	53.68	48.95^**(**)^	47.28^**(**)^	60.28	A/A A/G G/G	**0.0014** **0.0016** -	**0.0088** **0.0010** 0.9999	0.6234 0.3864 -
	[51.19-88.54]	[62.16-94.52]		[53.44-80.85]	[55.43-82.90]	[42.31-65.05]	[33.76-66.59]	[41.51-64.75]	[36.82-77.55]				
8-oxo2dG [ng/mL]	5.11	1.51	-	6.284^*^	6.260	6.809	2.126^*(***)^	**1.778**^(**)^	1.691	A/A A/G G/G	**0.0190** 0.7388 -	**0.0005** **0.0015** 0.3273	**0.0121** 0.1423
	[2.077-6.845]	[0.8240-9.117]		[4.974-8.311]	[4.824-9.118]	[3.376-10.24]	[1.353-4.325]	[0.8130-4.689]	[0.7760-6.036]				
OGG1 [ng/mL]	1.273	0.8670	-	1.651	1.723^*^	6.306	1.772	**1.208**^(**)^	1.682	A/A A/G G/G	0.1103 0.6216 -	0.8497 **0.0103** 0.3273	0.1656 **0.0346** -
	[0.6020-2.104]	[0.4440-1.762]		[0.9240-2.087]	[1.015-2.503]	[2.111-10.50]	[0.9190-2.443]	[0.6310-1.687]	[1.018-2.521]				
8-oxo2dG/OGG1	3.444	2.481	-	3.938	3.292	1.287	1.118^**(**)^	**1.744**^(*)^	1.642	A/A A/G G/G	**0.0040** 0.5662 -	**0.0054** **0.0480** 0.9091	0.8383 0.2942 -
	[1.660-6.107]	[1.072-7.585]		[1.6591 -6.493]	[2.489-5.788]	[0.9753-1.599]	[0.6668-3.102]	[0.7681-3.397]	[0.420-3.277]				

Median [1^st^-3^rd^ quartile]; Mann-Whitney test; ^*^p<0.05, ^**^p<0.01, ^***^p<0.001 as compared to unrelated controls, (^*^/^**^/^***^) p as compared to related controls.

## DISCUSSION

So far, the pathomechanism of AD has been explained by various hypothesizes [49], including: neurofibrillary degeneration [50], amyloid cascade [51], impaired Aβ clearance [52], *e.g.,* due to apolipoprotein misbalance [53, 54], neuroinflammation [55], mitochondrial dysfunction [13, 56, 57] and increased oxidative stress [58]. Factors responsible for generation of oxidative stress in AD include: Hcy [59], reduced antioxidant capacity [60], biometals, such as copper, iron, and zinc [61], disturbed glutamate signalling [62], microglia activation [63], as well as Aβ oligomers and hyperphosphorylated tau protein [64].

Dorszewska *et al.* [34] showed that in individuals over 60 years of age, the level of Hcy was significantly increased, probably due to decreased availability of cofactors essential for its metabolism, such as folic acid, and B vitamins [65]. The increased Hcy level may be seen in 25% of elderly persons without signs of dementia and may be associated with decreased cognitive functions [66]. According to the recent metaanlaysis of 111 papers by Setién-Suero [35], as well as Polish data [67–69], and our study, the Hcy levels were increased in the course of AD, possibly due to multiple mechanisms, which would include increased stress and neurotoxic agents.

Moreover, the level of Hcy in AD patients may depend on the advancement of the disease, as well as the used pharmacotherapy [35]. Our study has shown that Hcy level is particularly increased in the early stage of this dementive disease. Similar results were obtained by Kim *et al.* [70] and Sachdev *et al.* [71].

It seems that accumulation of Hcy in the central nervous system (CNS) may deteriorate function of the blood-brain-barrier (BBB) causing neurotoxic effects via damaging vascular endothelium and disturbed the production of nitrogen oxide [72]. Therefore, Hcy is thought to be one of the most significant factors associated with the risk of vascular and degenerative diseases, such as AD [73]. Peripheral Hcy concentration reflects its level in the brain, as Hcy crosses the BBB [74]. Fuso *et al.* [75], have shown that the elevated concentrations of Hcy in AD may lower the level of S-adenosylmethionine (SAM), essential for DNA methylation, thus may affect activity of presenilin 1 (PS1) and beta-site amyloid precursor protein cleaving enzyme 1 (BACE1), responsible for Aβ synthesis, associated with severity of the disease. In Polish population of AD patients, we showed that significantly higher level of Hcy may correspond to the severity of clinical symptoms.

Another mechanism of Hcy toxicity was described by Genedani *et al.* [59], who demonstrated that Hcy may interact as a NMDA receptors agonist, opening calcium ion channels and facilitating ion influx. Hcy mediated activation of NMDA receptor also induces decreased membrane potentials, and insufficient ATP production due to mitochondrial failure [76], visible by the release of cytochrome c [77]. The NMDA receptors are also a target for memantine, a drug commonly used for the treatment of AD, both single, or in combination with other drugs, for instance, AchE inhibitors. Memantine may act as neuroprotective agent [78] and may improve neuroplasticity in brain damaged by ischemia episodes [79]. Subsequently, both Gubandru *et al.* [80], and our studies have shown that treatment of AD patients with memantine or donepezil (AchEI) alone, or in combination, may be associated with a tendency for decreased Hcy as compared to non-treated patients, probably due to enhanced turnover, improved mitochondrial function and decreased oxidative stress [77].

It is known that 50% of Hcy is metabolized to Met, while the rest to Cys, used further for GSH synthesis [34, 81]. In AD, these metabolic pathways of the transformation of Hcy to Met and Cys are disturbed, possibly influencing the GSH production [34].

Subsequently, the data of the level of GSH in AD are divergent. Some data point out that GSH does not change significantly in *post-mortem* brains of AD patients [82] or may even rise in certain areas, including hippocampus [83], in the late stage of the disease. In turn, Gu *et al.* [84], and Venkateshappa *et al.* [85] reported the decreased concentration of GSH in the brains of AD patients. Interestingly, also the reduced level of plasma GSH may be observed in the early stage of dementia (MCI) [86, 87], and in AD patients, in whom the GSH concentration is reduced by roughly 13% [88], what is in line with our results. The reduced content of GSH is probably an effect of excessive Hcy production due to disturbed transformation to Cys [89].

According to our studies, the GSH level may depend on the advancement of the disease, as well as used pharmacotherapy. It would seem that the described relationship is the strongest in the early stage of the disease. On the other hand, particularly the therapy with memantine may lead to the improved transformation of Hcy to GSH, reduced ROS production as well as better mitochondria efficiency [81]. The GSH content decreases in severe dementia and the compensatory mechanisms associated with oxidative stress become disturbed.

It seems that reduction of GSH in AD may be associated with increased oxidative stress and may promote accumulation of DNA oxidation adducts, such as 8-oxo2dG [90]. The elevated level of 8-oxo2dG was observed in nuclear DNA from frontal, temporal, and parietal lobes and cerebellum of AD patients [91], as well as in peripheral tissues [34, 37, 40, 41]. However, Lovell *et al.* [92, 93], showed the decreased concentration of oxidized guanine in cerebrospinal fluid of AD patients. We observed similar differences in plasma of AD patients. Subsequently, it seems that presence of 8-oxo2dG in DNA during replication may lead to mutations and production of nonfunctional proteins, including DNA repair enzymes [38].

The main mechanism repairing oxidative damage in DNA by excising 8-oxo2dG depends on an OGG1 protein that seems to play a role both, in normal aging and in persons with an increased risk of developing AD. OGG1 content may be decreased in the brain [94], peripheral lymphocytes [40, 41], as well as in serum [37] of AD patients. In turn, in the present study we observed a reverse tendency for increase of OGG1 level in plasma of AD patients, what could be a response to the CNS DNA damage, as a consequence of the ongoing dementia process and the late activation of repair mechanisms. However, in conditions of developing the disease and increasing oxidative stress, the effectiveness of the enzyme is weakening, probably due to accumulated mutations and other genetic changes [95]. It seems that polymorphisms in *TOMM40* and *APOC1* genes may also contribute to excessive oxidative stress in persons predisposed for AD onset.

Our study showed that overrepresentation of *TOMM40*’523 L allele in the AD as well as the frequencies of other alleles, after adjusting for age, are in line with another Polish survey, by Maruszak *et al.* [24]. Although the literature data on *TOMM40*’523 are divergent, several reports indicate that the length variants (S, L, and VL) may be associated with the AOO of AD. Majority of reports, including the present study, show that the L variant is in strong linkage disequilibrium with *APOE* E4. Hence its independent effect is very difficult to analyze. The outcome of the remaining two alleles (S and VL) may be determined by several factors, including the presence of the *APOE* E4 allele, the age of the patients, familial, especially maternal, history of AD, the evaluated phenotype, the time of assessment and the length of follow up period [96]. The original work on *TOMM40*’523 variant by Roses *et al.* [26] indicated that in *APOE* E3/E4 carriers – VL allele may be associated with earlier symptoms of AD. Similar results were provided by subsequent works [22, 23, 97–99] and in part by Payton *et al.* [100] and Crenshaw *et al.* [101]. In turn, several works were not able to point out the role of *TOMM40* homopolymers in AD [102–105]. The only study on the Polish population, by Maruszak *et al.* [24], indicated the contrary, the VL variant to be more frequent in patients who developed AD after 79 years of age. Several of the newer studies, including our data, have shown that the *TOMM40*’523 S variant may be associated with a faster cognitive decline or earlier dementia onset [21, 106]. Similarly, the data on *APOE* E3/E3 homozygous persons presented by Payton *et al.* [100] and Crenshaw *et al.* [101] were parallel to our observations. Furthermore, a recent work of Willette *et al.* [107] pointed out that familial and especially maternal history and of AD have a significant impact on the effect of *TOMM40’*523 S and VL alleles on AOO and rate of cognitive decline, also in younger subjects. The VL genotype was accompanied with the faster cognitive decline, while the tendency reversed in favor of S genotype in older persons. Interestingly, the observed increased frequency of L allele in persons with family history of AD occurred probably due to its linkage to *APOE* E4.

The possible mechanism of *TOMM40*‘523 length polymorphism on AOO may be associated with changed TOM40 protein production in the brain. Linnertz *et al.* [108], reported that the *TOMM40*’523 VL/VL genotype was accompanied with increased levels of both proteins (apoE and TOM40) in the AD autopsy brains. Moreover, the authors were able to reproduce those results *in vitro* by luciferase assays on two cell lines: hepatoma HepG2 and neuroblastoma SH-SY5Y. Another study confirmed those observations using luciferase reporter, founding that the VL/VL genotype was accompanied with a doubled expression as of S/S variant [100]. It was shown that overexpression of TOM40 improves the mitochondrial function and efficiency, abolishes the Aβ mitochondrial toxicity [109], possibly by improving import of repair enzymes and antioxidants [110], produced in cytoplasm and transferred to mitochondria [111], via dicarboxylate carrier (DIC) [112] and TOM40 pores [113], which in AD may be blocked by Aβ oligomers [114].

The correlation between gene variants associated with the generation of oxidative stress via mitochondria (*TOMM40*), performed for the first time in AD patients in this study, indicated that presence of single or double VL allele (‘523) most likely leads to a reduction in GSH levels as a consequence of Hcy production. Subsequently, the decline of antioxidant potential leads to significant disruption of excision of oxidation adducts from DNA. It seems that in these patients the late manifestation of dementia symptoms was associated with an additional factor. However, the presence of two S/S alleles (‘523) led to a decrease in the efficiency of oxidative DNA damage repair systems (expressed as the ratio of 8-oxo2dG/OGG1) and earlier onset of the disease.

The *TOMM40*’650 SNP may also be engaged in the proper function of mitochondria and thus the generation of oxidative damage in AD. According to current reports, and the present study, the G allele of this SNP was overrepresented in AD [115] and was strongly connected with *APOE* E4 [15, 116]. This SNP may affect TOM complex, probably by introducing new splicing site, impeding the function of the TOM40 protein, hence decreasing the availability of antioxidant peptides (GSH) and repair proteins to mitochondria (OGG1). The polymorphism was also connected with dyslipidaemia and artery damage [117, 118]. For the first time, our analysis showed that in AD patients, either or both, A/A genotype (‘650) and VL allele (‘523) were associated with more pronounced Hcy generation at the expense of GSH, expressed in decreased GSH/Hcy ratio, and less effective excision of damaged forms of nucleotides (8-oxo2dG) from DNA. In addition, the major predisposition factor for dementia for Polish AD patients with A/A genotype (‘650) present in 67% of carriers, would comprise the elevated concentration of Hcy (above 15 μmol/L). Since only 56% of G/G carriers had increased Hcy, the cause of facilitated AD onset for this genotype would require additional studies.

Similarly, the effect of *APOC1*’638 SNP in AD may also be associated with the generation of oxidative stress. The frequency analyses in AD confirmed our findings as G allele is more frequent in dementia [7–9]. Furthermore, the allele remains in strong, but not absolute, linkage with *APOE* E4. Interestingly, this SNP is significantly associated with a cingulate Aβ load in the human brain [119] and with an increase of low density lipoproteins [120], hence alleviating the risk of atherosclerosis and BBB damage. To our knowledge present study for the first time correlates peripheral biothiols and oxidative stress markers in respect to this SNP. Our study showed that, in AD, the presence of either one or two A alleles (‘638) was associated with an increase in Hcy concentration, most likely at the expense of GSH. Additionally, the decrease of the natural antioxidant level most likely led to the generation of oxidative DNA damage and a decrease in the efficiency of the DNA repair system, especially when family relationships with AD patients were taken into consideration. On the other hand, in the patients with G/G genotype (‘638), another factor than the inordinate transformation of biothiols and the deficiency of the DNA repair system was probably responsible for early dementia onset. Nevertheless, the explanation of the *APOC1*’638 dependent mechanism in AD pathogenesis requires further studies.

What is worth noting, the combining of data on the genetic status of *APOE* E4, *TOMM40*’650 and *APOC1*’623 increases significantly the sensitivity of assessing the risk of developing late onset AD based solely on *APOE* status [6, 115, 121, 122]. Moreover, the AD brain is under the influence of excessive oxidative stress, as soon as in preclinical stage [123], it may enhance appearing of symptoms, thus explaining why the patients with inordinate stress responses due to *TOMM40* and *APOC1* rare variants may develop AD in younger age [124, 125].

## METHODS

### Subjects

The study was subjected to 230 individuals. 88 AD patients, diagnosed according to NINCDS-ADRDRDA criteria [126] were enrolled in the study. The 10.3% of patients were early diagnosed, prior treatment, 57.3% of patients received acetylcholinesterase inhibitors (AchEI: donepezil or rivastigmine, in doses 5-10 mg/daily or 3-6 mg/daily, respectively), 11.8% of patients were treated with memantine (5-20 mg/daily) and 20.6% of patients were treated with combination of memantine and donepezil (10-20 and 5-10 mg/daily). We also recruited 80 control volunteers over 60 years of age with no signs of dementia and other neurological disorders or family history of AD. The comparative group comprised 62 volunteers aged > 60 years with a positive family history of AD and no signs of dementia or other neurological diseases. The alleles of the *APOE* gene were analyzed in all subjects. The demographic data are shown in Table 7. All participants or their legal guardians provided signed a written consent. The research project was approved by the Bioethical Committee at the Poznan University of Medical Sciences, a decision no. 1031/13, dated May 5, 2013, with subsequent supplement.

**Table 7:** Alzheimer’s disease and control’s demographic and genetic data, including age, sex distribution, *APOE* frequencies, duration of the disease, mini-mental (MMSE) score and used pharmacotherapy

Parameters		Unrelated controls (UC)	Related controls (RC)	Alzheimer’s disease (AD)
No. of cases [n]		80	62	88
Age [years]^*^		70 [60–90]	64 [60–78]	76 [60–94]
Sex [% female]		78.8	72.6	67.0
*APOE* genotypes [%]	E2/E2	1.3	0.0	0.0
	E2/E3	13.8	9.7	3.4
	E2/E4	3.8	1.6	3.4
	E3/E3	68.8	45.2	42.0
	E3/E4	12.5	40.3	43.2
	E4/E4	0.0	3.2	8.0
Duration of the disease [%, years]	<5	-	-	67.9
	>5	-	-	32.1
MMSE score		Within normal range [27–30]		15.3±6.7^#^
Treatment [%]	None	100	100	10.3
	AchEI	0	0	57.3
	Mem	0	0	11.8
	AI+M	0	0	20.6

^*^Median [min-max], ^#^Mean±SD, otherwise percent values

AchEI – acetylcholinesterase inhibitors, Mem – memantine, AI+M – acetylcholinesterase inhibitors + memantine.

### Material

Each participant’s blood was collected on an anticoagulant – K_3_EDTA (Monovette™ vacuum system, Sarstedt, USA). A total volume of 3 ml of blood was immediately aliquoted, frozen and stored at -80°C upon nucleic acid isolation (Gravity Flow, A&A Biotechnology, Poland). Subsequently, the remaining blood was centrifuged (1400 RCF, 10 min). The obtained plasma was aliquoted and stored at -80°C until further processing.

### Homocysteine and glutathione quantification

The concentrations of plasma biothiols (Hcy and GSH) were analyzed by High Performance Liquid Chromatography with Electrochemical Detection (HPLC/EC) (Dionex, Germany/ESA, USA) according to Dorszewska *et al.* [34]. Briefly, 150 μL of plasma was diluted with 75 μL of water. Subsequently, the thiols were reduced with 25 μL of 10% TCEP (tris[2-carboxyethyl]phosphine) in water. Then, the sample was deproteinized with 500 μL 1M HClO_4_ and centrifuged for 5 min at 12,000 RCF, and the supernatant was injected into HPLC/EC system equipped with C18 column (250 mm x 4.6 mm x 5 μm) with pre-column (ThermoFisher Scientific, Germany) and eluted with aqueous buffer supplied with 25 mM NaH_2_PO_4_, 15 mM sodium dodecyl sulphate and 17% acetonitrile (vol.) at pH=2.90. The data were integrated and analyzed by Chromelon software.

### OGG1 and 8-oxo2dG quantification

Determination of the plasma 8-oxo2dG and OGG1 concentrations was performed by the ELISA method. For 8-oxo2dG, the assay measured free circulating nucleotide in the plasma, excised from DNA, without prior DNA extraction and digestion. Similarly, the assay for OGG1 measured free circulating protein. The analysis was performed according to the manufacturer’s protocols (EI LAB, China) on undiluted plasma samples. Absorbance was measured by the EPOCH microplate reader (BioTek, USA). The concentrations were calculated from a four-parametric standard curve (R=0.998) by Gen5 ver. 2.01 software (provided with the reader). Inter- and intra-assay coefficients of variability amounted: 5.3% and 7.2% for 8-oxo2dG and 6.5% and 9.7% for OGG1, respectively.

### APOE genotyping

The method of *APOE* genotyping has been described previously [127]. Briefly, the 40-50 ng of DNA extracted from frozen blood (Genomic Micro AX Blood Gravity, A&A Biotechnology, Poland) was used for Real-Time qPCR (CFX Connect, 1x SsofastEvaGreenSupermix, Bio-Rad, USA) with three pairs of primers (c=6.25 mM) corresponding to three common alleles of *APOE* (E2, E3, and E4, respectively). The reactions with Cq≤28 (≤10 cycle of secondary qPCR) were considered positive.

### TOMM40’523 genotyping

For the carriers of *APOE* E4 allele, the *TOMM40*‘523 variant was analyzed by modified capillary electrophoresis method described before [24]. Briefly, 40-50 ng of genomic DNA was amplified with 10 mM corresponding primers (forward labeled with 5-FAM), Hi-Fi polymerase in a mixture of 1x Hi-Fi buffer, 1.6x Amplifier and 4 mM dNTPs (Novazym, Poland). The electrophoresis was carried out by an external laboratory on ABI3000 analyzer (GE, USA). The data was analyzed by Peak Scanner software. The results were validated by sequencing by an external laboratory.

Subsequently, for the remaining subjects (without *APOE* E4 allele), with a bimodal distribution of S and VL T*OMM40*‘523 variants, the analysis was performed by High Resolution Melting Analysis (HRM), according to: Laczó *et al.* [19], with modifications. Briefly, 20 ng of genomic DNA was used for Real-Time-qPCR (CFX Connect, 1x SsofastEvaGreenSupermix, Bio-Rad, USA) with corresponding primers (c=6.25 mM), next, the melting analysis was performed, and the data was analyzed by Melting Analysis software (Bio-Rad, USA). The HRM results were cross-referenced with the data obtained by capillary electrophoresis and validated by direct sequencing of 10% of samples in a commercial facility.

***TOMM40’650 and APOC1’638 genotyping*** were performed by HRM method in conditions described above, and similarly, 10% of samples were validated by direct sequencing in an external laboratory.

The cycling conditions of PCRs and HRM, as well as the sequences of the primers used, shall be provided upon request.

***The statistical analysis*** was performed using GraphPad for Windows software and Statistica 12.5 for Windows (StatSoft, USA), with the nonparametric Kruskal-Wallis and Mann-Whitney tests, unless stated otherwise. The normality of data distributions was checked using the Shapiro-Wilk test (when the sample size were 50 or less) and Kolgomorov-Smirnow (when the sample size is larger than 50). Analysis of the homogeneity of variance was carried out using the Levene’s test. Considering the potential false positive rate incurred by multiple comparisons of several gene associations in control and two patient groups, we applied the Bonferroni correction method to adjust the p value (p_corr_). The level of significance was set as p<0.05.

## CONCLUSIONS

Out of the analyzed variants, the *TOMM40*’523-L and *APOC1*’638-G were in the strongest linkage disequilibrium with *APOE* E4, while the association was much less pronounced for *TOMM40*’650-G SNP, especially in persons without a family history of AD. In the course of AD, the VL and A alleles of the analyzed genes (*TOMM40*’523 and *APOC1*’650) were associated with the generation of Hcy and with a decrease in the formation of antioxidants (GSH), followed by the reduced level of oxidized nucleotide (8-oxo2dG) removed from DNA. On the other hand, in patients with *TOMM40*’523 S/S genotype, most likely reduced the efficiency of the oxidative damage repair system (OGG1) led to the accumulation of oxidative damage in DNA and early symptoms of dementia.

## SUPPLEMENTARY MATERIALS TABLES







## Abbreviations

8-oxo2dG: 8-oxo-2’-deoxyguanosine
Aβ: amyloid β
AchEI: acetylcholinesterase inhibitor
AD: Alzheimer’s disease
apoC1: apolipoprotein C1
apoE: apolipoprotein E
AOO: age of onset
BACE1: beta-site amyloid precursor protein cleaving enzyme 1
BBB: blood-brain barrier
BER: base excision repair
CNS: central nervous system
Cys: cysteine
GSH: glutathione
Hcy: homocysteine
HHcy: hyperhomocysteinemia
HPLC/EC: high pressure liquid chromatography with electrochemical detection
HRM: high resolution melting
MCI: mild cognitive impairment
Met: methionine
MMSE: Mini–Mental State Examination
mtGSH: mitochondrial glutathione
OGG1: 8-oxoguanine DNA glycosylase
PS1: presenilin 1
RC: related controls - control volunteers with positive family history of Alzheimer’s disease
ROS: reactive oxygen species
SAM: S-adenosylmethionine
SNP: single nucleotide polymorphism
TOM40: translocase of the outer mitochondrial membrane homolog 40
UC: unrelated controls: control volunteers without positive family history of Alzheimer’s disease
