# Supplementary material for: Biothiols and oxidative stress markers and polymorphisms of TOMM40 and APOC1 genes in Alzheimer’s disease patients
Source: Oncotarget. 2018 Oct 16;9(81):35207–25. doi: 10.18632/oncotarget.26184 (PMC6219666; doi:10.18632/oncotarget.26184)
Supplement: Supplementary file 1 [file oncotarget-09-35207-s001.pdf]

Biothiols and oxidative stress markers and polymorphisms of *TOMM40* and *APOC1* genes in Alzheimer's disease patients

SUPPLEMENTARY MATERIALS

Supplementary Table 1: The concentration of homocysteine (Hcy), glutathione (GSH), 8-oxo-2'-deoxyguanosine (8-oxo2dG) and 8-oxoguanine DNA glycosylase (OGG1) in plasma of Alzheimer's disease (AD) patients stratified according to dementia severity and related (RC) and unrelated controls (UC).

See Supplementary File 1

Supplementary Table 2: The concentration of homocysteine (Hcy), glutathione (GSH), 8-oxo-2'-deoxyguanosine (8-oxo2dG) and 8-oxoguanine DNA glycosylase (OGG1) in plasma of Alzheimer's disease (AD) patients stratified according to used drug treatment and related (RC) and unrelated controls (UC).

See Supplementary File 2

Supplementary Table 3: The concentration of homocysteine (Hcy), glutathione (GSH), 8-oxo-2'-deoxyguanosine (8-oxo2dG) and 8-oxoguanine DNA glycosylase (OGG1) in plasma of Alzheimer's disease (AD) patients stratified according to duration of the disease and related (RC) and unrelated controls (UC)

Group	Unrelated controls (UC)	Related controls (RC)	AD <5 years	AD >5 years	p			
Parameter					K-W	Group	*vs.* UC$^{@}$	*vs.* RC$^{@}$
Hcy [μmol/L]	13.1<br>[10.6-17.5]	13.2<br>[10.8-16.5]	**17.7**$^{*(*)}$<br>[11.4-20.8]	**17.3**$^{*(*)}$<br>[12.5-21.2]	**0.0079**$^{\$}$<br>0.6239$^{\#}$	<5 years<br>>5 years	**0.0162**<br>**0.0222**	**0.0120**<br>**0.0140**
GSH [μmol/L]	910.0<br>[782.5-1116]	887.1<br>[764.6-1055]	**828.5***<br>[681.0-1064]	**778.0***<br>[717.5-952.5]	**<0.0001**$^{\$}$<br>0.8513$^{\#}$	<5 years<br>>5 years	**0.0368**<br>**0.0371**	0.1531<br>0.1227
GSH/Hcy	73.0<br>[52.2-88.6]	68.5<br>[53.4-82.7]	**54.6**$^{***(**)}$<br>[38.69-74.43]	**42.9**$^{**(**)}$<br>[35.47-58.66]	**<0.0001**$^{\$}$<br>0.4319$^{\#}$	<5 years<br>>5 years	**0.0003**<br>**0.0011**	**0.0019**<br>**0.0017**
8-oxo2dG [ng/mL]	5.016<br>[1.576-7.081]	6.284<br>[4.956-8.692]	**1.655**$^{**(***)}$<br>[0.9548-4.285]	**2.931***<br>[1.811-6.246]	**<0.0001**$^{\$}$<br>0.2308$^{\#}$	<5 years<br>>5 years	**0.0043**<br>0.3355	**<0.0001**<br>**0.0151**
OGG1 [ng/mL]	1.211<br>[0.5765-2.101]	1.706<br>[1.002-2.503]	**1.417**$^{(*)}$<br>[0.6893-2.045]	1.329<br>[0.9933-3.052]	**0.0447**$^{\$}$<br>0.4166	<5 years<br>>5 years	0.5973<br>0.1552	**0.0440**<br>0.7464
8-oxo2dG/OGG1	3.257<br>[1.635-6.204]	3.298<br>[1.868-6.170]	**1.379**$^{**(***)}$<br>[0.6336-3.165]	2.540<br>[0.8656-4.305]	**0.0032**$^{\$}$<br>0.4841	<5 years<br>>5 years	**0.0022**<br>0.1823	**0.0008**<br>0.1391

Median [1st-3rd quartile]; $^{\$}$-Kruskal-Wallis test (all groups); $^{\#}$-Mann-Whitney test (AD patients groups); $^{@}$-Mann-Whitney test; $^{*}p<0.05$, $^{**}p<0.01$, $^{***}p<0.001$ as compared to unrelated controls, (*/**/***) p values as compared to related controls; AD – Alzheimer's disease.

Supplementary Table 4: Genetic variants and age of onset of Alzheimer's disease

Loci	Alzheimer's disease (AD) Age of onset [years]						p values	
	**S/S**	**S/VL**	**VL/VL**	**S/L**	**L/L**	**L/VL**	*vs.* VL/VL$^{@}$	
							ANOVA	0.1014
							S/S	**0.0418**
TOMM40'523	**68.57*** ±9.361	72.10 ±8.154	76.43 ±6.925	**71.00*** ±7.021	**68.60**** ±5.103	**71.43*** ±5.854	S/VL	0.1121
							S/L	**0.0370**
							L/L	**0.0062**
							L/VL	**0.0492**
	**A/A**		**A/G**		**G/G**		$p^{\#}$	
							A/A vs G/G	0.0804
TOMM40'650	71.78 ±7.955		73.19 ±7.026		**68.58**$^{(*)}$ ±4.582		A/G vs G/G	**0.0219**
							A/A vs A/G	0.4392
	**A/A**		**A/G**		**G/G**		$p^{\#}$	
							A/A vs G/G	**0.0153**
APOC1'638	72.47 ±9.146		72.50 ±5.788		**67.42**$^{*(**)}$ ±4.502		A/G vs G/G	**0.0076**
							A/A vs A/G	0.9999
		**VL/A/A**			**L/G/G**		$p^{@}$	
Major vs minor alleles		82.00 ±3.464			**68.29**** ±5.282		**0.0013**	
		**E2/E3+E3/E3**			**E4/E4**		$p^{@}$	
APOE		72.74 ±8.525			**65.57*** ±6.630		**0.0408**	

Mean±SD, $^{@}$-unpaired *T* test, $^{\#}$-unpaired *T* test with Welch correction; $^{*}p<0.05$, $^{**}p<0.01$, as compared major genotype; (*/**) p as compared to A/G.
