# Supplementary material for: Biothiols and oxidative stress markers and polymorphisms of TOMM40 and APOC1 genes in Alzheimer’s disease patients
Source: Oncotarget. 2018 Oct 16;9(81):35207–25. doi: 10.18632/oncotarget.26184 (PMC6219666; doi:10.18632/oncotarget.26184)
Supplement: Supplementary file 2 [file oncotarget-09-35207-s002.docx]

Supplementary Table 1: The concentration of homocysteine (Hcy), glutathione (GSH), 8-oxo-2’-deoxyguanosine (8-oxo2dG) and 8-oxoguanine DNA glycosylase (OGG1) in plasma of Alzheimer’s disease (AD) patients stratified according to dementia severity and related (RC) and unrelated controls (UC)

| Group  Parameter | Unrelated controls (UC) | Related controls (RC) | MCI | Mild dementia | Moderate dementia | Severe dementia | p | | | | |
| --- | --- | --- | --- | --- | --- | --- | --- | --- | --- | --- | --- |
|  |  |  |  |  |  |  | K-W | Group | *vs.* UC^@^ | *vs.* RC^@^ |  |
| Hcy [µM] | 13.1  [10.6-17.5] | 13.2  [10.8-16.5] | 20.3  [12.2-20.7] | **17.7^*(*)^**  [11.65-21.50] | 16.0  [11.48-19.3] | **19.4^**(**)^**  [14.80-21.55] | **0.0162^$^**  0.4362^#^ | **MCI**  **Mild**  **Mod**  **Sev** | 0.2995  **0.0459**  0.2172  **0.0052** | 0.2660  **0.0330**  0.1529  **0.0040** |  |
| GSH [µM] | 910.0  [782.5-1116] | 887.1  [764.6-1055] | 691.0  [651.0-830.0] | **832.5^*^**  [664.8-969.0] | 822.5  [733.5-1092] | **772.0^*^**  [711.3-892.5] | 0.0756^$^  0.5139^#^ | **MCI**  **Mild**  **Mod**  **Sev** | 0.0598  **0.0498**  0.4921  **0.0269** | 0.1205  0.1307  0.8908  0.0748 |  |
| GSH/Hcy | 73.0  [52.2-88.6] | 68.5  [53.4-82.7] | **54.4^*(*)^**  [40.11-56.64] | **51.1^***(**)^**  [37.43-71.53] | 59.0  [43.00-82.50] | **41.3^***(***)^**  [29.88-59.47] | **<0.0001^$^**  0.1061^#^ | **MCI**  **Mild**  **Mod**  **Sev** | **0.0156**  **0.0007**  0.1230  **0.0006** | **0.0276**  **0.0023**  0.2054  **0.0010** |  |
| 8-oxo2dG [ng/mL] | 5.016 [1.576-7.081] | 6.284  [4.956-8.692] | 3.525  [3.225-6.874] | **1.282^**(***)^**  [0.869-1.696] | **2.125^(***)^**  [0.996-4.366] | **2.931^(*)^**  [2.194-5.749] | **<0.0001^$^**  0.0560^#^ | **MCI**  **Mild**  **Mod**  **Sev** | 0.8417  **0.0016**  0.0535  0.6838 | 0.4652  **<0.0001**  **0.0006**  **0.0388** |  |
| OGG1 [ng/mL] | 1.211  [0.5765-2.101] | 1.706  [1.002-2.503] | 1.318  [1.312-1.360] | 1.508  [0.7043-2.223] | **1.098^(*)^**  [0.6170-1.830] | 1.565  [0.9665-2.246] | 0.1197^$^  0.6006^#^ | **MCI**  **Mild**  **Mod**  **Sev** | 0.7390  0.2777  0.8506  0.1912 | 0.2368  0.3668  **0.0433**  0.7498 |  |
| 8-oxo2dG/ OGG1 | 3.257  [1.635-6.204] | 3.298  [1.868-6.170] | 2.675  [2.371-3.730] | **0.723^***(***)^**  [0.5696-1.7320] | **1.846^(*)^**  [0.8898-3.186] | 2.567  [1.0749-5.432] | **0.0022^$^**  0.0806^#^ | **MCI**  **Mild**  **Mod**  **Sev** | 0.9621  **0.0003**  0.0853  0.4546 | 0.7954  **0.0002**  **0.0410**  0.3388 |  |

Median [lower-upper quartile]; $ -Kruskal-Wallis test (all groups); # - Kruskal-Wallis test (AD groups); @ - Mann-Whitney test; *p<0.05, **p<0.01, ***p<0.001 as compared to unrelated controls; (*/**/***) p values as compared to related controls

MCI – mild cognitive impairment; Mild – mild AD, Mod – moderate AD, Sev – severe AD
