# Supplementary material for: Biothiols and oxidative stress markers and polymorphisms of TOMM40 and APOC1 genes in Alzheimer’s disease patients
Source: Oncotarget. 2018 Oct 16;9(81):35207–25. doi: 10.18632/oncotarget.26184 (PMC6219666; doi:10.18632/oncotarget.26184)
Supplement: Supplementary file 3 [file oncotarget-09-35207-s003.docx]

Supplementary Table 2: The concentration of homocysteine (Hcy), glutathione (GSH), 8-oxo-2’-deoxyguanosine (8-oxo2dG) and 8-oxoguanine DNA glycosylase (OGG1) in plasma of Alzheimer’s disease (AD) patients stratified according to used drug treatment and related (RC) and unrelated controls (UC)

| Treatment | Unrelated controls (UC) | Related controls (RC) | Alzheimer’s disease patients (AD) | | | | p | | | |
| --- | --- | --- | --- | --- | --- | --- | --- | --- | --- | --- |
|  |  |  | Non-treated | AChEI | Memantine | AChEI+  Memantine |  |  |  |  |
|  |  |  |  |  |  |  | K-W | Group | *vs.* UC^@^ | *vs.* RC^@^ |
| Hcy [µM] | 13.1  [10.6-17.5] | 13.2  [10.8-16.5] | **22.9^**(***)^**  [20.3-24.4] | 14.9  [11.0-19.5] | 17.0  [12.3-21.5] | **18.9^**(**)^**  [16.3-20.7] | **0.0014^$^**  0.0636^#^ | N  AI  M  AI+M | **0.0020**  0.3186  0.0898  **0.0079** | **0.0007**  0.2224  0.0878  **0.0048** |
| GSH [µM] | 910.0  [782.5-1116] | 887.1  [764.6-1055] | 790.0  [671.0-935.5] | **772.0^**(*)^**  [683.5-896.3] | **1091.5^(*)^**  [993.3-1153] | 755.5  [693.3-1067] | **<0.0001^$^**  0.0656^#^ | N  AI  M  AI+M | 0.1873  **0.0042**  0.1094  0.1955 | 0.2428  **0.0180**  **0.0308**  0.3623 |
| GSH/Hcy | 73.0  [52.2-88.6] | 68.5  [53.4-82.7] | **40.0^**(***)^**  [28.5-52.5] | **51.5^**(*)^**  [38.3-75.8] | 65.5  [56.8-82.5] | **42.9^***(***)^**  [41.6-57.8] | **<0.0001^$^**  0.0956^#^ | N  AI  M  AI+M | **0.0013**  **0.0034**  0.5373  **0.0007** | **0.0005**  **0.0100**  0.9926  **0.0009** |
| 8-oxo2dG [ng/mL] | 5.016 [1.576-7.081] | 6.284  [4.956-8.692] | **1.629^(**)^**  [1.125-4.293] | **2.110^(*)^**  [1.036-7.635] | **2.059^(*)^**  [0.8410-3.916] | **1.865^**(***)^**  [1.071-3.035] | **<0.0001^$^**  0.6908^#^ | N  AI  M  AI+M | 0.1613  0.3026  0.1080  **0.0061** | **0.0080**  **0.0102**  **0.0109**  **<0.0001** |
| OGG1 [ng/mL] | 1.211  [0.5765-2.101] | 1.706  [1.002-2.503] | 1.843  [1.320-2.126] | 1.397  [0.7570-1.878] | 1.998  [0.6985-2.408] | 1.387  [0.7038-2.195] | 0.1596^$^  0.8322^#^ | N  AI  M  AI+M | 0.2414  0.6154  0.3184  0.3792 | 0.8618  0.1057  0.7805  0.4512 |
| 8-oxo2dG/  OGG1 | 3.257  [1.635-6.204] | 3.298  [1.868-6.170] | **0.8072^*(*)^**  [0.7233-2.313] | 1.642  [0.6379-5.598] | 1.474  [0.4842-3.386] | **1.618^*(**)^**  [0.8532-2.943] | **0.0100^$^**  0.7464^#^ | N  AI  M  AI+M | **0.0430**  0.1128  0.0811  **0.0137** | **0.0305**  0.0651  0.0709  **0.0052** |

Median [lower-upper quartile]; $-Kruskal-Wallis test (all groups); #-Kruskal-Wallis test (AD patients groups); @- Mann-Whitney test; *p<0.05, **p<0.01, ***p<0.001 as compared to unrelated controls, (*/**/***) p values as compared to related controls

N – nontreated patients, AChEI (AI) – acetylcholine esterase inhibitors, M – memantine, AI+M – acetylcholine esterase inhibitors + memantine
